# Research on Vehicle Trajectory Deviation Characteristics on Freeways Using Natural Driving Trajectory Data

**DOI:** 10.3390/ijerph192214695

**Published:** 2022-11-09

**Authors:** Zhenhua Dai, Cunshu Pan, Wenlei Xiong, Rui Ding, Heshan Zhang, Jin Xu

**Affiliations:** 1 College of Traffic & Transportation, Chongqing Jiaotong University, Chongqing 400074, China; 2 CCCC Second Highway Consultant Co., Ltd., Wuhan 430056, China; 3 Chongqing Key Laboratory of “Human-Vehicle-Road” Cooperation and Safety for Mountain Complex Environment, Chongqing Jiaotong University, Chongqing 400074, China

**Keywords:** traffic engineering, traffic safety, driving behavior, geometric alignment, lane width, trajectory deviation

## Abstract

Lateral driving behavior analysis is the foundation of freeway cross-section design and the focus of road safety research. However, the factors that influence vehicle lateral driving behavior have not been clearly explained. The dataset of the natural driving trajectory of freeways is used in this study to analyze vehicle lateral driving behavior and trajectory characteristics. As vehicle trajectory characteristic indicators, parameters such as preferred trajectory deviation and standard deviation are extracted. The effects of lane position, speed, road safety facilities, and vehicle types on freeway trajectory behavior are investigated. The results show that lane width and lane position significantly impact vehicle trajectory distribution. As driving speed increases, the lateral distance between vehicles in the inner lane and the guardrail tends to increase. In contrast, vehicles in the outside lane will stay away from the road edge line, and vehicles in the middle lane will stay away from the right lane dividing line when the speed increases. Statistical analysis shows that the preferred trajectory distribution of the same vehicle type in different lane positions is significantly different among groups (Cohen’s d > 0.7). In the same lane, the lateral position characteristics of the center of mass of different vehicle types are basically the same (Cohen’s d < 0.35). This work aims to explain what variables cause trajectory deviation behaviors and how to design traffic safety facilities (guardrail and shoulder) and lane width to accommodate various vehicle types and design speeds.

## 1. Introduction

Recent years have seen an increase in light-duty vehicles, represented by passenger cars, vans, SUVs, and pickup trucks. Many cities in China have begun to build light freeways (special freeways for cars), such as Shenzhen’s Jihe freeway and Fujian’s Quanxia freeway. This measure has proven to be an effective way to take into account traffic safety, comfort, and resource conservation. It can prevent the reduction in road capacity and serious traffic conflicts caused by traditional mixed traffic. Lane width, as a critical indicator of light freeway cross-section design, not only meets the safety needs of vehicle overtaking and moving side by side, but also meets the psychological safety distance required by drivers from the road environment. As a result, on the one hand, a thorough understanding of the lateral driving behavior, lateral position preference, and influencing factors of vehicle trajectories can assist some cities in designing and building light freeways by providing data support and specification preparation basis. On the other hand, it can reveal the mechanism of freeway traffic accidents, which is useful for researching freeways accident prevention strategies.

Table 1 shows the lane width specifications provided by several industrialized countries’ freeway construction standards and regulations [1,2,3,4,5,6]. The distribution range of the lane width is 3.5~3.9 m. For example, China’s Freeway Route Design Specification (JTG D20—2017) [1] stipulates that the lane width is 3.75 m when the design speed of the freeway and Class-A road (arterial road that access can be controlled as needed) is 80 km/h or above. It can be found that the specified lane width in most countries is based on the cross-sectional layout of mixed traffic mode [2,3,4,5,6], where the key technical indicators are controlled by large vehicles (2.55 m in body width of representative model), taking into account the swing offset and lateral margin of vehicles in the lane, so the lane width has a large surplus relative to the body width, especially for cars (1.8 m in body width of representative model). In a lane keeping scenario, drivers have more freedom in trajectory selection, and the vehicles deviate laterally from the lane’ s centerline, resulting in trajectory selection preferences. In addition to design redundancy, it will also lead to accidents related to lane deviation, such as side impact, rear-end collisions, and run-off-road crashes. Therefore, a thorough understanding of drivers’ trajectory selection behavior is the basis of lane width design and also helps to improve road safety conditions.

Previous research has primarily focused on the impact of lane width on traffic flow characteristics. Peng et al. [7] proposed a lattice point model to describe the effect of lane width lateral separation on traffic flow, used numerical simulation to show the evolution mechanism of lane width on traffic congestion and discovered that the lane width effect has an impact on the stability of traffic flow. Zheng et al. [8] used urban traffic flow data to demonstrate that there was no statistical correlation between capacity and lane width while increasing the number of lanes and the right lateral clearance had a significant effect on capacity. Tang et al. [9] proposed a multi-lane traffic flow model considering lane width, number of lanes, and lane shift, suggest that when the traffic density of each lane was low or high, the change in lane width was significant. Furthermore, some researchers found that lane width can influence vehicle speeds. Based on road data, Chitturi et al. [10] argued that the reduction in lane width and lateral clearance lead to a decreased in vehicle free flow speed. Typically, the narrower the lane, the greater the deceleration, and heavy vehicles are more affected than cars. Fitzpatrick et al. [11] analyzed the impact of lane and shoulder width configuration on vehicle operation and created a lane speed prediction model based on cross-section geometric indicators. The results show that when the lane width is increased from 3.4 to 3.7 m, the lane speed will increase by about 3.5 km/h, and the increase in the left shoulder width will also increase the lane speed. Kondyli et al. [12] recorded traffic flow data under various road conditions. They found that reducing lane width and shoulder width had a significant impact on the free flow speed, and the free flow speed decreased by 1 mile per hour for every foot drop. Lane width also affects driving behavior, Wei [13] proposed the mechanism of different lane reduction methods (including unilateral and bilateral reduction) on driving behavior characteristics by driving simulation scenes and explaining the lane reduction effect using psychological load theory. The results showed that narrowing the lanes not only reduced the running speed, but also had a significant impact on the choice of driving lateral position. Wang et al. [14] used high simulation driving simulation equipment to compare the effects of different widths of inner lane on the running of light-duty vehicles in tunnel scenes. The difference in lane widths of 3.75 and 3.5 m was found to have no significant effect on speed and trajectory deviation. Mecheri et al. [15] investigated the effect of simulated road section redistribution on driving behavior using a driving simulator. The work discovered that as lane width narrows, vehicles move closer to the center of the road. The shoulder width was increased, the vehicle was moved closer to the lane edge line, and they proposed the tolerant design concept of widening the shoulder to provide a lane recovery area in the event of a wrong turn by the driver. Liu et al. [16] used a tunnel simulator to test the effect of lane width, lane position, and shoulder width on lane deviation. The examine found that vehicles driving in the lane on the side of the wall would stay away from the wall, and the wide shoulder could reduce the proportion of dangerous lateral displacement and improve driving comfort and safety.

For nearly two decades now, statistical methods have been used to investigate the impact of road cross-sectional parameters on driving safety or to develop accident prediction models [17]. Wood et al. [18] conducted a correlation analysis between accident data and roadway geometric conditions and discovered that the presence or absence of curb strips was a significant parameter in the accident statistical model. Ashraf et al. [19] suggest that no correlation between the width of a two-lane freeway and crash frequency based on characteristics statistics of traffic accidents. Wu et al. [20] quantified the safety performance of freeways under different lane width conditions, and the accident statistics indicate that the standard size (mean width of 3.45 m) had the lowest crash probability, while either narrow lanes (mean width of 3.25 m) or wider lanes (mean width of 3.75 m) increased the crash frequency. Dixon et al. [21] investigated vehicle operation data collected on urban freeways and discovered that increasing left shoulder width can reduce the likelihood of traffic safety accidents, in other words, increasing shoulder width contributes more to traffic safety than increasing lane width. According to an accident prediction model, Zegeer et al. [22] argued that lane width and shoulder width have significant effects on freeway safety, but incremental lane width and shoulder width have a decreasing marginal effect. The sum of curvature changes, number of circle curve turns, and speed limit all have a positive relationship with the number of head-on collisions, according to Zhang et al. [23], whereas variables such as lane and shoulder width have no effect on the incidence of head-on collisions.

In summary, most previous studies focused on single-factor correlation analysis, and the majority of the data collected were cross-sectional observation data, accident data, driving simulator collection data, and computer simulation data in regular roads, which ignored the fact that the natural running of vehicles was a continuous process and could not reveal the influence mechanism of real road environment, lane position, and vehicle types on driving behavior, which would affect the research’s accuracy. It cannot provide reliable evidence for the cross-section design and revision of standards and specifications of special freeways for cars without the verification of massive vehicle trajectory data, and the risk assessment method by using accident data cannot reveal the mechanism of traffic safety accidents on freeways.

In this paper, the preferred trajectory deviation is proposed for the first time to characterize lateral driving behavior using the natural driving trajectory dataset of German freeways. The trajectory behavior analysis in a wider and higher speed range is carried out in the straight section of freeway. This work aims to investigate how lane position, vehicle type, and vehicle speed affect trajectory deviation. It distinguishes the differences in center of mass between cars and trucks. Clarification of vehicle trajectory behavior characteristics and influencing factors can provide a valuable theoretical basis for determining key technical indicators of freeway cross-section and road safety facilities such as guardrail and shoulder, thereby reducing the drivers’ psychological load.

## 2. Data Sources and Research Methods

### 2.1. The HighD Dataset

The HighD Dataset, a drone dataset of naturalistic vehicle trajectories on German freeways, is used as the data source for freeway vehicle lateral position analysis in this paper [24]. The recordings took place during sunny and windless weather from 8:00 a.m. to 5:00 p.m. A total of 11.5 h of aerial video with a frame rate of 25 Hz was recorded, 110,000 single continuous vehicle trajectory is extracted (including approximately 20,000 trucks). The total mileage of vehicles recorded was 45,000 km, including 5600 complete lane change records. The position error is typically less than 10 cm when using state-of-the-art machine learning algorithms. Center of mass position (trajectory point) and movement of the vehicles appearing in each frame are recorded from a bird’s eye view, including detailed information such as vehicle size, vehicle type, driving direction, speed, and description of surrounding vehicles.

### 2.2. Observation Sites

**Figure 1:**
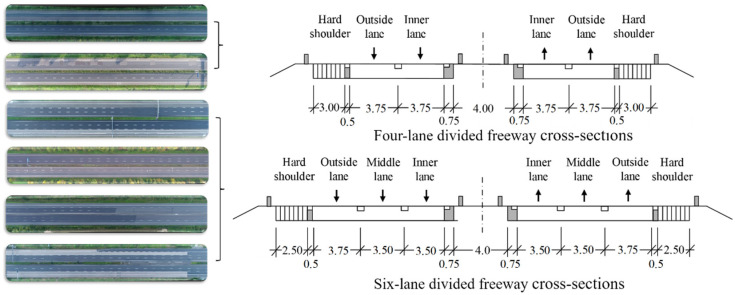
Illustration of the cross-section at the HighD dataset collection sites (unit: m).

**Figure 2:**
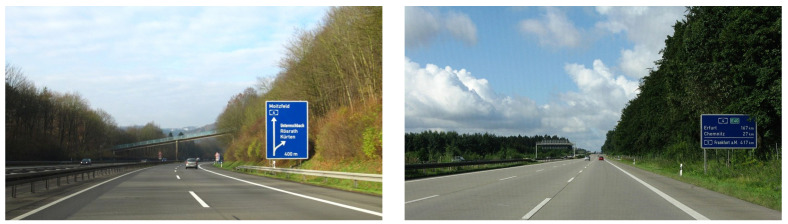
Guardrail and roadside environment at the collection site.

The HighD dataset was recorded on straight freeway sections in six different locations near Cologne, Germany. There were two or three lanes in each direction. The shooting range on the straight sections is approximately 420 m. Figure 1 and Figure 2 depict the collection locations of roadside environment and cross-sectional form. The lane width on the four-lane divided freeway is 3.75 m, the left marginal strip is 0.75 m, the right marginal strip is 0.5 m, and the right hard shoulder width is 3 m. The outside lane on a six-lane divided freeway is 3.75 m wide, the middle and inner lanes are 3.5 m wide, the left marginal strip is 0.75 m wide, the right marginal strip is 0.5 m wide, and the right hard shoulder is 2.5 m wide. The medium strip is 4 m long, the guardrail is a W-beam guardrail, and natural vegetation covers the outside of the right shoulder. The widths of standard cars and trucks are 1.8 and 2.5 m, respectively.

**Figure 3:**
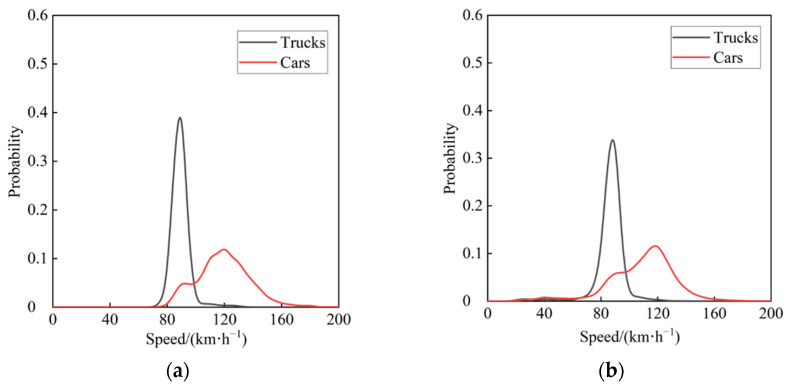
(**a**) Speed distribution on four-lane divided freeways; (**b**) speed distribution on six-lane divided freeways.

Because some German freeways have no speed limits, the trajectory data of a wider speed range can be observed, and analysis of its trajectory behavior characteristics can provide key indicators for the cross-section of freeways at different design speeds. Figure 3a depicts the vehicle speed distribution ranges on the four-lane divided freeways in the highD dataset, where trucks’ speeds are concentrated in 80–90 km/h and car speeds are primarily distributed 90–150 km/h. Figure 3b depicts the vehicle speed distribution ranges on the six-lane divided freeways in the highD dataset. Truck speed distribution is primarily 70–95 km/h, while car speed distribution is primarily 80–140 km/h. Furthermore, in Germany, vehicles with a gross weight of less than 3.5 t are not restricted in lane use; vehicles with a gross weight greater than 3.5 t drive in the right lane, whereas trucks with a gross weight greater than 3.5 t typically drive in the outside lane. Those that are 3.5 t typically drive in the outside lane.

## 3. Trajectory Behavior Characteristic Indicators

**Figure 4:**
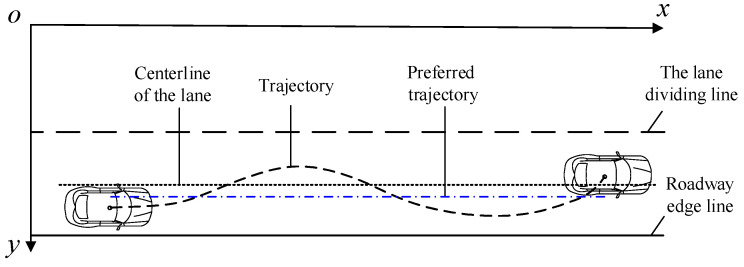
Illustration of feature parameters.

Lane keeping is among the most common events on the freeway. The natural driving behavior of vehicles in straight sections is observed, It is discovered that drivers will not strictly keep the center of the vehicle in the lane centerline, but use the lane width to keep the vehicle in the lane. In other words, the vehicle trajectory point deviates from the lane centerline and swings. Therefore, this paper proposes the preferred trajectory, the preferred trajectory deviation, and the preferred trajectory standard deviation index to describe the lane vehicle’s trajectory behavior characteristics. The parameter calculation is shown in Figure 4. The preferred trajectory is the mean value of the ordinate of all trajectory points in the vehicle’s lane, which reflects the vehicle preferred lateral position, and the trajectory usually swings around it. The lateral distance between the vehicle’s preferred trajectory and the lane’s centerline when the lane is kept is referred to as the preferred trajectory deviation, reflecting the driver’s preference for trajectory selection. The standard deviation of the preferred trajectory represents the dispersion degree of all vehicle preferred trajectories in the same lane.

**Figure 5:**
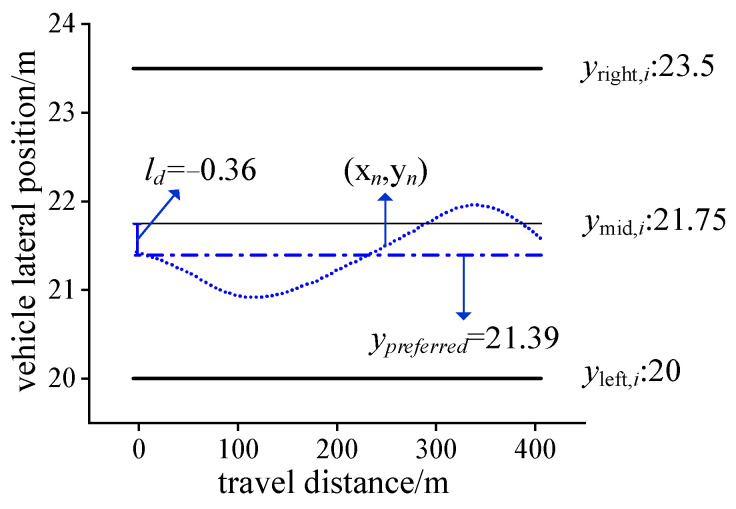
Example of calculation of feature parameters.

The preferred trajectory characteristic parameters of the concrete calculation method are shown in Figure 5. Create a rectangular coordinate system with the upper left corner of the aerial video as the coordinate origin, and the lanes are numbered from 0 to *i* in ascending order. Lane markings run horizontally in the image. Therefore, their positions can be indicated by a *y*-coordinate, where *y_left,i_* is the longitudinal coordinate of lane *i*’s left lane marking; *y_mid,i_* is is the longitudinal coordinate of lane *i*’s centerline; and *y_right,i_* is the longitudinal coordinate of lane *i*’s right lane marking in the coordinate system. The vehicle trajectory is excluded if it is not recorded by the aerial video throughout the process. Then, the trajectories with the number of lane changes of 0 will be taken as the object. The coordinates of the trajectory point (*x_n_*, *y_n_*) and the coordinates of the road marking are superimposed in the same coordinate system to obtain the trajectory-road marking topology relationship. The average value of the longitudinal coordinates of the trajectory point is the preferred trajectory, and the formula for calculating the preferred trajectory is shown in Equation (1). The distance between the preferred trajectory and the centerline of the lane is the preferred trajectory deviation, and the formula for calculating the preferred trajectory deviation is shown in Equation (2).

(1) $$y_{preferred}=\frac{1}{n}(y_{1}+y_{2}+\cdot \cdot \cdot +y_{n})$$

(2) $$l_{d}=y_{preferred}-y_{mid,i}$$

where *n* is the number of trajectory points; *y_n_* is the longitudinal coordinate of the vehicle’s the *n*th trajectory point; *y_preferred_* is the preferred trajectory, representing the driver’s lateral preference position in the lane; *l_d_* is the preferred trajectory deviation. When the preferred trajectory falls on the left of the centerline of the lane, the preferred trajectory deviation is negative. Otherwise it is positive. In this study, the 15th and 85th percentile of the preferred trajectory are used as the feature numbers to show the drivers’ lane utilization.

## 4. Analysis of Factors Influencing the Preferred Trajectory Deviation

### 4.1. Preferred Trajectory Distribution Characteristics

Driving behavior in the lane is the result of psychological load, environmental perception, and road geometric characteristics. The preferred trajectory represents the driver’s preferred position in different lane environments and speeds. It can aid in understanding the mechanism of driving behavior as well as the impact of multiple factors on driver trajectory selection behavior.

**Figure 6:**
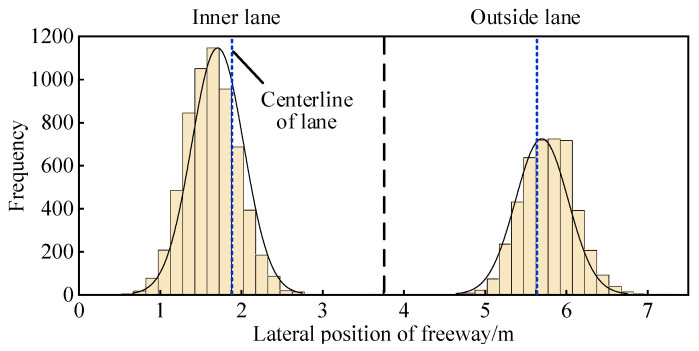
Preferred trajectory distribution on four-lane divided freeways.

**Figure 7:**
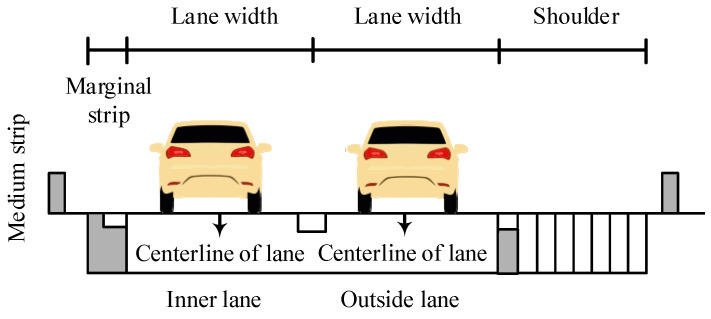
Illustration of the preferred trajectory preference position profile on four-lane freeways.

Figure 6 depicts a histogram of the preferred trajectory deviation distribution for four-lane divided freeways, with the blue dashed line representing the lane centerline and the black dashed line representing the lane boundary line. The characteristic value of preferred trajectory deviations for four-lane divided freeways is presented in Table 2. The average preferred trajectory deviation of the inner lane is 1.710 m, accounting for 31.12% of the trucks, and the average preferred trajectory deviation of the outside lane is 5.706 m, accounting for 44.27% of the trucks. It shows left and right deviation from the lane centerline, respectively. Figure 7 represents the lateral position preference of the vehicle in the lane using the peak value of the preferred trajectory distribution, demonstrating that during natural driving, the preferred trajectory of the vehicle in the same direction tends to one side close to the lane edge line, implying that the presence of the shoulder will cause the preferred trajectory to deviate. Furthermore, there is no significant difference in the dispersion degree of lanes on both sides, indicating that when the lane width is the same in the four-lane divided freeways scene, there is no significant difference in the preferred trajectory dispersion degree.

**Figure 8:**
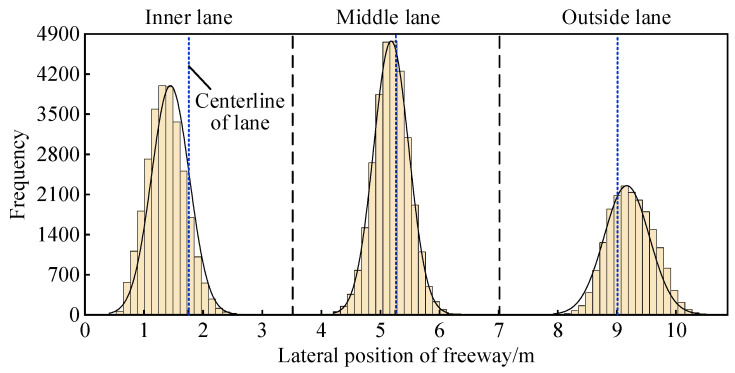
Preferred trajectory distribution of six-lane divided freeways.

**Figure 9:**
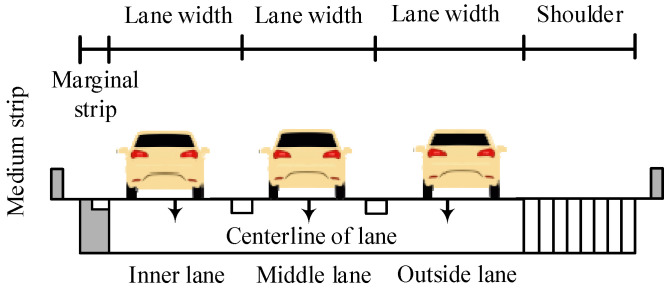
Illustration of the preferred trajectory preference position profile on six-lane freeways.

The preferred trajectory deviations of the six-lane divided freeways are depicted in Figure 8 and Table 3. The mean values of the preferred trajectory deviations from the inner lane to the outside lane are 1.423, 5.185, and 9.175 m, respectively, indicating left, left, and right deviations. Figure 9 characterizes the lateral position preference of the vehicle within the lane using the peak of the preferred trajectory distribution, indicating that in the natural driving state, the preferred trajectory of the vehicle in the inner lane and outside lane tends to move to the side of roadway edge line, with the behavior of riding on the markings and shoulders. The preferred trajectory of the middle lane tends to stay away from the side of right lane dividing line. This can be explained by the presence of marginal strips and shoulders on both sides of the lane, which provide lateral safety clearance and extra space for vehicles in the lane, and the deviation position keeps a larger distance between the parallel vehicles in the same direction, which is conducive to reducing the driver’s psychological load. In contrast, the vehicles moving in the middle lane need to be parallel with the trucks in the outside lane. Due to the trucks’ large body size and large mass, the drivers perceive a greater safety risk than cars, so they will adjust the vehicle’s position to maintain a wider distance from the heavy vehicles in the outside lane.

The dispersion of the outside lane of the six-lane divided freeway road (3.75 m) is greater than that of the inner and middle lanes (3.5 m). The dispersion degree of the middle lane is smaller than that of the inner lane, indicating that the wider the lane, the more lateral space is provided for vehicles, and the dispersion degree of the trajectory will also increase. Moving vehicles are on both sides of the driver in the middle lane. To avoid collisions with parallel vehicles traveling in the same direction, a clear lateral distance will be maintained with both sides, and the preferred trajectory will be limited, resulting in a lower dispersion degree than that of the same width lane. Furthermore, when traveling in parallel with trucks, drivers perceive greater safety risks than small cars, resulting in a greater lateral distance between them. As a result, the lane width, and the environment (including the types of neighboring vehicles and roadside facilities) on both sides of the lane influence vehicle lateral driving behavior.

To investigate the influence on vehicle preferred trajectory deviation in different scenarios, the preferred trajectory distributions of the inner and outside lanes of the four-lane divided freeway and six-lane divided freeway were compared and analyzed to investigate the differences in lateral driving behavior of the same lane location in different scenarios, as well as to compare the breakdown of the major vehicle types in the lanes.

**Figure 10:**
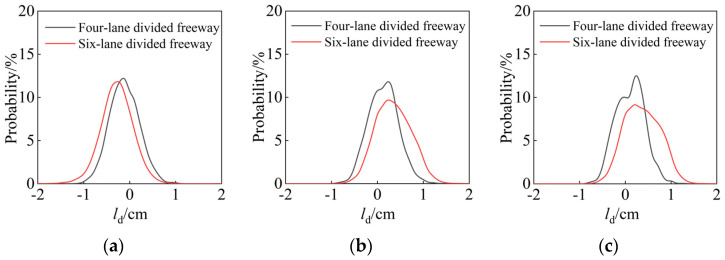
(**a**) Preferred trajectory distribution of the inner lane of cars; (**b**) preferred trajectory distribution of the outside lane; (**c**) preferred trajectory distribution of the outside lane of trucks.

As shown in Figure 10a, the mean and peak of the preferred trajectory deviation of cars in the six-lane divided freeway lane are greater than those in the four-lane divided freeway lane, and drivers are more inclined to drive close to the roadway edge line. Still, the distribution pattern is not significantly different. Figure 10b depicts the preferred trajectory distribution of the outside lane in two scenarios: a six-lane divided freeway versus a four-lane divided freeway, with the six-lane divided freeway having more significant right deviation distance from the centerline. The probability distribution curves of trucks in the outside lanes of two scenes are superimposed in Figure 10c. The probability distribution curve of four-lane divided freeway trucks has two peaks, and the probability distribution curve of six-lane divided freeway trucks has a right side deviation, indicating that, when compared to four-lane divided freeway vehicles, the center of mass of six-lane divided freeway vehicles prefers to be located on the right while driving in the outside lane. This could be due to the fact that the width of the adjacent lanes in the six-lane divided freeway scene is narrower than in the four-lane divided freeway scene, which interferes with truck lateral driving behavior to some extent, and truck drivers tend to keep a larger distance.

### 4.2. The Effect of Car Speed on the Preferred Trajectory Deviation

To determine the critical index values of cross-section facilities required by light freeways at different design speeds, it is necessary to explore the trajectory control characteristics of car drivers at different speeds. This section selects the preferred trajectory deviation of passenger cars at different speeds for comparison. This section compares the preferred trajectory deviation of cars at various speeds. The neighborhood of 10 times in the range of 100–160 km/h is chosen as the speed retrieval condition, and the neighborhood’s floating threshold is −3–3 km/h. The sample size is approximately 300 vehicles under different speeds. Figure 9 and Figure 10 depict the relationship between car speed and the preferred trajectory deviation on four-lane divided freeways and six-lane divided freeways. The straight line where *y* = 0 is located represents the centerline of the lane. The preferred trajectory deviation is positive when *l_d_* falls on the right side of the centerline of the lane. Otherwise, it is negative.

**Figure 11:**
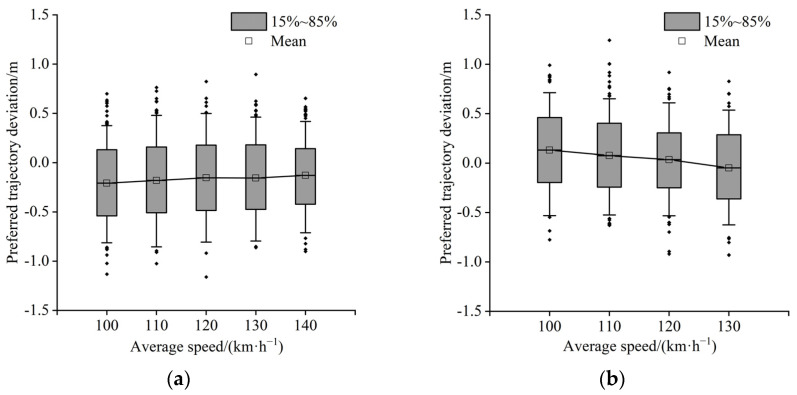
(**a**) Inner lane; (**b**) outside lane.

**Figure 12:**
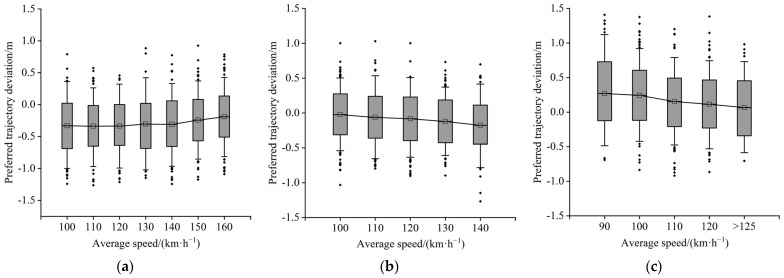
(**a**) Inner lane; (**b**) middle lane; (**c**) outside lane.

Figure 11 and Figure 12 show the relationship between speed and the preferred trajectory deviation of a four-lane divided freeway and a six-lane divided freeway, respectively. Figure 11a and Figure 12a show that as driving speed increases, the lateral distance between the vehicle in the inner lane and the left guardrail tends to increase, indicating that the demand for the vehicle’s lateral safety margin increases as well. Furthermore, the preferred trajectory of the middle lane continues to move to the left (Figure 12b). Figure 12b,c show that the outside lane tends to drive towards the centerline of the lane, and the preferred trajectory distribution range in the inner and middle lanes also tends to shrink.

The change in trajectory control behavior after speed increase can be explained by the “field of safety” theory. Gibson et al. [25] suggest that drivers adjust their speed and lane position according to the perceived “field of safe travel”. If they deviate from the safe field while running, it will cause tension or increase anxiety, and drivers’ constant behavior adjustment is to keep their running state within an acceptable range. The field force on the vehicle in its actual driving state represents the level of safety risk associated with the vehicle. The greater the safety risk, the greater the field force, and the field force is primarily determined by the potential field strength, the vehicle’s motion state, and the vehicle’s attributes.

As the vehicle’s speed increases, so does its original field force, and the energy and harm caused by collision with the guardrail is greater, so the vehicles in inner lane will tend to stay away from the median guardrail. The virtual “external force” generated by the vehicle in the outside lane, particularly the truck, increases, forcing the vehicle in the middle lane to change its motion state during acceleration and move laterally to the left to obtain more safe lateral space. Furthermore, the potential field strength (constraint effect) of the road boundary field is increased, and vehicles in the outside lane are more willing to continue driving in the center of the lane. As a result of the increased speed, vehicles in the inner lane tend to be further away from the guardrail, and the lane should be adjusted to the outer side (right side). Namely, after increasing the design speed, the width of the left marginal strip should be increased moderately.

### 4.3. The Effect of Vehicle Types on the Preferred Trajectory Deviation

**Figure 13:**
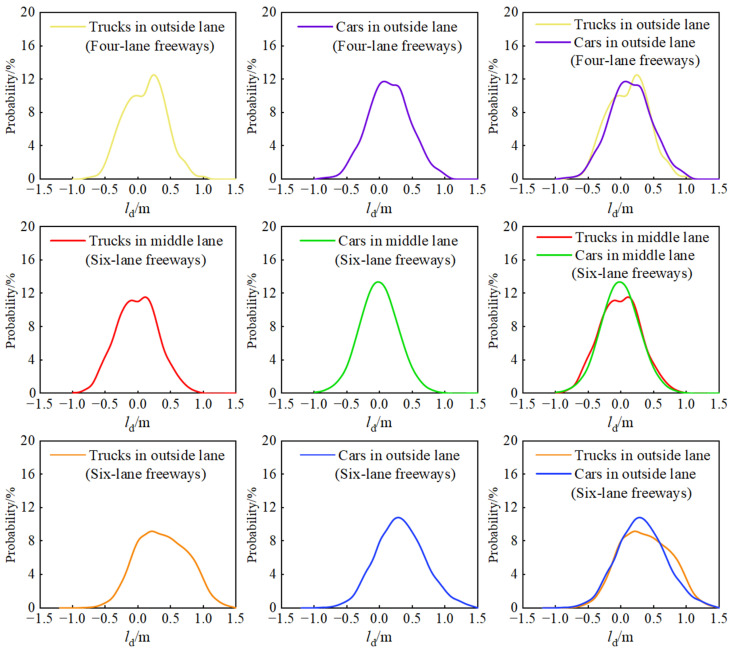
Distribution of trajectory deviation of different vehicle types.

To clarify the differences between the operating characteristics of light-duty vehicles and large trucks, and to derive the key indicators of freeway suitable for light-duty vehicles, the motion behavior of both must be analyzed. To ensure that lane position and roadside environmental variables do not affect vehicle trajectory characteristics, the objects for comparative analysis are the mixed passenger and freight lane in two cross-sectional forms. Figure 13 shows probability curve combination diagrams of the preferred trajectory deviation of various vehicles in the lane, and Table 4 shows the statistical characteristic values of the preferred trajectory deviation of various types of vehicles in the lane.

Table 4 shows that there is no discernible difference between the mean and standard deviation of the preferred trajectory of different vehicle types in the same lane. Therefore, the intuitive probability distribution curve does not accurately reflect the influence of various vehicles on the preferred trajectory deviation. To test the influence of vehicle types on trajectory deviation, statistical tools should be used. A pairing test refers to the effect of research factors on the results when samples are compared in pairs. It is a commonly used statistical tool to test for a significant difference between two types of samples.

The T-distribution theory is used to infer the probability of difference, so as to compare whether there are significant differences between the two data groups. Statistical method of paired T distribution is calculated, the differences in trajectory control between different vehicle types in the same lane and the same type in different lanes are compared, and the significance of vehicle types and lane width is analyzed. Among them, when the *p* value of the test result is less than 0.05, the statistical result is significant, indicating that there is a significant difference between them. Cohen’s d is the effect size that indicates the difference between groups. The values 0.20, 0.50, and 0.80 correspond to small, medium, and large effect critical points.

Statistical analysis in Table 5 shows that the preferred trajectory deviation of different types of vehicles when driving in lanes differs significantly. However, according to Cohen’s d value, the difference between the two types of vehicles in the outside lanes is small, and the difference between the middle lanes is also small. It demonstrates that the lateral position characteristics of the center of mass of different types in the same lane are essentially the same. Given the difference in body width between the two types of vehicles, it is reasonable to conclude that lane width used by cars is lower than trucks. Therefore, the currently specified lane width value for light-duty vehicles can be reduced. To clarify the degree of lane width utilization of different vehicle types under lane keeping conditions, the *eigenvalues* of preferred trajectory distribution was selected from 10th to 90th, and the actual lane width occupied by different types in this distribution range was calculated. For straight road sections with lane widths of 3.5 and 3.75 m, the usage lane widths of trucks are 3.3 and 3.6 m, and the usage lane widths of cars are 2.5 and 2.8 m.

There are significant differences when comparing the same vehicle type’s preferred trajectory deviation distribution in the outside lane (3.75 m) and the middle lane (3.5 m). The difference between trucks in different lane is medium, and the difference between cars in different lane is large. This demonstrates that changes in the roadside environment and lane width result in different trajectory behavior characteristics of the same type of vehicle, and car drivers are more sensitive to perception and response to the surrounding environment.

## 5. Discussion

This research differs from previous single-factor research in that it considers many key factors of the “human-vehicle-road” collaborative safety system. The data source employed is large-scale driving trajectory data, which is more accurate than data collected by driving simulation technology. The research findings are more credible when applied to engineering design. According to current research [15], the presence of a shoulder affects the trajectory deviation direction, and the reduction in lane width reduces the vehicle preferred trajectory deviation distance. Statistics show that when different types of vehicles share the same lane, the difference in center position between cars and trucks is small, and the difference between preferred trajectory distribution and body width can be used to design lane width for different types of vehicles. Furthermore, it is stated in the [16] that the lane position of the underground freeway will affect vehicle deviation, but the direction of deviation is opposite to the research result of this paper. It could be because the dark and narrow driving environment increases psychological load, and drivers will keep a wider distance from the inner wall of the tunnel, proving that the roadside environment is among the key factors influencing lane deviation.

Meanwhile, this study found some interesting conclusions that were not found in previous studies:

When the vehicle’s speed increases, so do the virtual external forces that act on it. Guardrails, parallel vehicles, and road markings can provide virtual forces, and the force increases with speed. This aids in understanding the mechanism of driving behavior and its relationship to speed and road facilities, as well as providing a theoretical foundation for geometric layout and road safety protection measures at higher design speeds. For example, setting a wider distance between the inner lane and guardrail when design speed is improved. Additionally, increasing the width of the middle lane contributes to a better sense of security.

However, we must add a word of caution. The differences in driving styles cannot be ignored and should be investigated further. Further, other common road geometric features that need to be considered include circular curves with various radii and slopes. Another limitation could be the lack of bridge or tunnel scenes in the dataset. To strengthen the findings, future research should look into potential differences in driving styles, individual characteristics, geometric alignment, and traffic facilities.

## 6. Conclusions

This study analyzed the influences of lane width, lane position, road facilities, vehicle types, and speed on lateral driving behavior and trajectory control characteristics by using open natural driving trajectory dataset. The main findings can be summarized as follows:

(1) The preferred trajectory deviation is affected by lane position and the roadside environment. The preferred trajectory of the vehicle tends to be close to the roadway edge line when there is a shoulder or marginal strip on the outside of the lane; the middle lane tends to stay away from the trucks.
(2) By narrowing the lane, the preferred trajectory’s dispersion can be limited. Furthermore, lane position and roadside facilities influence the preferred trajectory dispersion, and the vehicle trajectory dispersion in the middle lane is lower than in the other two lanes.
(3) As speed increases, the distance between vehicles in the inner lane and the guardrail increases, and the vehicle preferred trajectory in the middle lane continues to move to the side away from the truck, while vehicles driving in the outside lane tend to close the lane’s centerline; thus, as the design speed increases, the width of the marginal strip should be appropriately increased to meet the demand of drivers’ psychological safety.
(4) The preferred trajectory deviation of the same type of vehicle in different lanes is noticeably and significantly different. Car drivers are more sensitive to their surroundings’ perception and reaction than truck drivers.
(5) According to statistical analysis, lane position and roadside environment are significant factors influencing the vehicle preferred trajectory deviation. When different types of vehicles are mixed in the same lane, the difference between the center positions of cars and trucks is small; in other words, the lateral position characteristics of the center of mass of cars are essentially the same as those of trucks, which can be used to justify lane width reductions on light freeways.

## Figures and Tables

**Table 1:** The specified of lane width of freeway in some countries (unit: m).

Road Class	China	USA	Germany	UK	Japan	Canada	Russia
Freeway	3.5~3.75	3.6~3.9	3.5~3.75	3.65~3.7	3.5~3.75	3.5~3.7	3.75
Urban freeway	3.5~3.75	3.6~3.9	3.5	3.65~3.7	3.5	3.7	3.75

**Table 2:** Characteristic value of preferred trajectory deviations for four-lane divided freeways.

Lane	Centerline (m)	Mean (m)	Median (m)	Standard Deviation	15th (m)	85th (m)	Sample Size	Speed (km/h)
Inner lane	1.875	1.710	1.702	0.322	1.379	2.046	6180	121.39
Outside lane	5.625	5.706	5.707	0.324	5.364	6.034	4322	96.52

**Table 3:** Characteristic value of preferred trajectory deviations for four-lane divided freeways.

Lane	Centerline (m)	Mean (m)	Median (m)	Standard Deviation	15th (m)	85th (m)	Sample Size	Speed (km/h)
Inner lane	1.75	1.423	1.427	0.349	1.081	1.773	28,103	87.20
Middle lane	5.25	5.185	5.184	0.299	4.881	5.491	30,119	108.06
Outside lane	8.875	9.175	9.156	0.383	8.77	9.594	19,074	117.64

**Table 4:** Statistical characteristic value of preferred trajectory deviation for different vehicle types.

Road Segment	Vehicle Types	Mean (m)	Standard Deviation	Median (m)	10th (m)	90th (m)	Sample Size
Four-lane divided freeway outside lane	trucks	0.058	0.314	0.076	−0.355	0.440	1922
	cars	0.099	0.332	0.088	−0.318	0.527	2401
Six-lane divided freeway middle lane	trucks	0.064	0.320	−0.066	−0.484	0.344	2330
	cars	0.065	0.297	−0.066	−0.438	0.312	27,789
Six-lane divided freeway outside lane	trucks	0.319	0.386	0.303	−0.169	0.837	12,052
	cars	0.268	0.376	0.253	−0.210	0.752	7022

**Table 5:** Difference test of preferred trajectory deviation for different vehicle types.

Road Segment	t Value	*p* Value	Cohen’s d Value
Four-lane divided freeway outside lane	−2.507	0.012 **	0.081
Six-lane divided freeway middle lane	0.000 ***	−10.351	0.334
Six-lane divided freeway outside lane	0.237	−1.183	0.038
Trucks in middle and outside lanes	0.000 ***	23.224	0.749
Cars in middle and outside lanes	0.000 ***	30.081	0.994

***, ** respectively represent the significance level of 1% and 5%.

## Data Availability

The HighD dataset is available online at: http://www.highD-dataset.com (accessed on 25 July 2022).
